# Examining the density in out-of-pocket spending share in the estimation of catastrophic health expenditures

**DOI:** 10.1007/s10198-021-01316-x

**Published:** 2021-08-06

**Authors:** Abdulrahman Jbaily, Annie Haakenstad, Mizan Kiros, Carlos Riumallo-Herl, Stéphane Verguet

**Affiliations:** 1 Department of Global Health and Population, Harvard T.H. Chan School of Public Health, Boston, MA USA; 2 Ethiopian Health Insurance Agency, Addis Ababa, Ethiopia; 3 School of Economics, Erasmus University, Rotterdam, The Netherlands

**Keywords:** Financial risk protection, Out-of-pocket health expenditures, Catastrophic health expenditures, Universal health coverage, Equity, I11, I13, I14, I15, I31, I32

## Abstract

Universal health coverage (UHC) aims to provide access to health services for all without financial hardship. Moving toward UHC while ensuring financial risk protection (FRP) from out-of-pocket (OOP) health expenditures is a critical objective of the Sustainable Development Goal for Health. In tracking country progress toward UHC, analysts and policymakers usually report on two summary indicators of lack of FRP: the prevalence of catastrophic health expenditures (CHE) and the prevalence of impoverishing health expenditures. In this paper, we build on the CHE indicator: we examine the distribution (density) of health OOP budget share as a way to capture both the magnitude and dispersion in the ratio of households’ OOP health expenditures relative to consumption or income at the population level. We illustrate our approach with country-specific examples using data from the World Health Organization’s World Health Surveys.

**Supplementary Information:**

The online version contains supplementary material available at 10.1007/s10198-021-01316-x.

## Introduction

In 2015, the United Nations General Assembly set a list of 17 Sustainable Development Goals (SDGs) to be achieved by its member states by 2030. The SDGs include 169 targets related to poverty, education, health, and many other social objectives. In particular, SDG 3, the SDG for Health, aims to “ensure healthy lives and to promote well-being for all irrespective of age, gender or location” and “to achieve universal health coverage including financial risk protection for all” [1, 2].

On the path toward fulfilling the SDGs, many countries and national governments have embarked on health system reforms aspiring to universal health coverage (UHC). Therefore, governments and international agencies routinely report on two key indicators constructed by analysts to monitor progress toward UHC: a health services coverage indicator, and a financial risk protection (FRP) indicator. First, the coverage indicator aims to assess whether people in need of health services receive those services with sufficient quality; and a composite index was recently formulated to track this coverage indicator nationally [3–5]. Second, the FRP indicator aims to quantify the proportion of households that are protected from the financial risks associated with out-of-pocket (OOP) expenditures upon seeking health services. Two indicators are commonly used to estimate the extent of lack of FRP at the country level: the prevalence of catastrophic health expenditures (CHE), and the prevalence of impoverishing health expenditures (IHE). On the one hand, OOP health expenditures are considered “catastrophic” when they exceed a certain fraction (e.g. $10\%$) of total household consumption expenditures or income [6, 7]. On the other hand, OOP health expenditures are considered “impoverishing” when they push total household consumption/income below a defined poverty line (e.g. international poverty line of $\$1.90$ per day, Purchasing Power Parity) [8–10]. Recently, utilizing a large collection of household surveys, Wagstaff and colleagues [11, 12] estimated CHE and IHE, in 133 and 122 countries, respectively, for the years 2000, 2005 and 2010. Estimating CHE and IHE enables the identification of country-level determinants of poor FRP, including, for example, high amounts of OOP expenditures, total health expenditures, government and prepaid share of total health expenditures, types of disease burden, and national income [7, 11–14].

The proposed objectives (by 2030) for the two UHC indicators of health services coverage and FRP are: all people should receive coverage of essential health services, and national health systems should provide full financial protection from OOP health expenditures [1]. Therefore, given the objective of universality, reporting on these two indicators requires employing metrics which can not only synthesize the average score in these indicators but also can represent their distributions at the country level. In addition, such UHC metrics should be easily interpretable and amenable to determining the underlying reasons behind potentially high/low scores, so that effective population-level interventions can be subsequently enacted. The routinely used CHE metric attempts to address this need for FRP. It uses a threshold of household consumption or income (e.g. 10–$40\%$), as a stepping stone toward producing easily computable and reproducible estimates of (lack of) FRP across and within countries.

In this paper, we propose a methodological approach that builds on existing measures of magnitudes of OOP health expenditures and CHE and examines their underlying distributions (magnitude and dispersion) at the population level. In the “Methods” section, we examine the full density of health OOP budget share as a way to capture both the magnitude and dispersion in the ratio of households’ OOP health expenditures relative to income (at the population level); and we discuss its main features. The “Results” section illustrates our approach with country-specific examples using data from the World Health Organization’s World Health Surveys. This section is intended as an application to our proposed methodological approach and is not meant to explicitly discuss the state (and findings) of the included countries. Finally, we conclude by highlighting the strengths and limitations of our CHE density approach and outline research areas for future work.

## Methods

### Examining the distribution of OOP budget share

We directly build on the indicator of CHE. When estimating CHE, analysts examine the fraction of a household’s consumption expenditures or income that is spent on OOP health expenditures. They compare the ratio between such OOP expenditures (the numerator) and household consumption expenditures or income (the denominator) to a certain pre-defined threshold (the “catastrophic” threshold). In what follows, we denote $\mathcal {F}$ such a ratio, which corresponds to the household health OOP budget share. Household consumption expenditures or income can be the denominator of $\mathcal {F}$ and we refer to it as household “income”. This is meant for conciseness of the semantics used in this paper, and it has no bearings on our analysis: the same mathematical formulations presented below can be derived exchangeably with consumption expenditures instead of income. The selection of income vs. consumption expenditures will be ultimately determined by the data at hand, where either one or both might be available. Likewise, our mathematical derivations can be implemented for the examination of individuals’ OOP expenditures (rather than households’). Also, in some studies, income can capture total earnings, while in others, essential expenditures such as subsistence expenditures (e.g. food consumption) can be subtracted from total earnings before the computation of the ratio $\mathcal {F}$ (OOP budget share). Again, these variations in definitions have no bearings on our mathematical exploration.

**Fig. 1:**
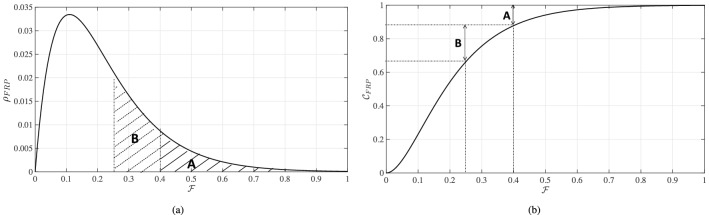
**a** Graphical representation of the density function $\rho _{\text{FRP}}$ of $\mathcal {F}$ (OOP budget share), and of its relationship to the prevalence of catastrophic health expenditures (CHE). Area A represents the proportion of the population facing CHE when a catastrophic threshold of $\tau =40\%$ is used; and area (A+B) when a catastrophic threshold of $\tau =25\%$ is used. ** b** Graphical representation of $\mathcal {C}_{\text{FRP}}$, the cumulative distribution function of $\rho _{\text{FRP}}$

We propose to focus on the state of distributional OOP budget share (both magnitude and dispersion of OOP budget share) in a given country. We refer to this as “distributional OOP share”, and denote it $\mathcal {D}_{\text{FRP}}$. Distributional OOP share is composed of two parts: first, a graphical representation (curve) of how OOP spending relative to income is distributed across a whole country population (denoted $\mathcal {C}_{\text{FRP}}$); and, second, a numerical index that utilizes the curve’s characteristics to provide a quantitative summary of the OOP share state (denoted $\mathcal {I}_{\text{FRP}}$). We construct the two parts of $\mathcal {D}_{\text{FRP}}$ by computing first the density function $\rho _{\text{FRP}}$ of the fraction $\mathcal {F}$ (Fig. 1a).

Figure 1a presents the variation in the density of $\mathcal {F}$ across a given population^1^. This is the empirical density function of the OOP budget share of income. The CHE summary metric can be directly estimated from $\rho _{\text{FRP}}$ once a catastrophic threshold is selected (e.g. 10, 25, or 40$\%$). For example, when a threshold of 40$\%$ is selected, area A (filled with solid lines) would represent the fraction of the population incurring CHE. When a threshold of $25\%$ is selected, however, area B (filled with dashed lines) would be added to area A, so the total area A+B would then represent the proportion of the population incurring CHE. The number of households experiencing any level of OOP spending can be easily computed using $\rho _{\text{FRP}}$ by finding the corresponding area under the curve and multiplying it by the total population. For example, the number of households that spend between 25 and $40\%$ of income on health would be equal to area B multiplied by the size of the population under study. This can be summarized by using the corresponding cumulative distribution function ($\mathcal {C}_{\text{FRP}}$), which constitutes the first part of our distributional approach (Fig. 1b). This is the empirical cumulative distribution function of the OOP budget share of income^2^. The proportion of households for whom OOP spending exceeds a selected catastrophic threshold (on such a curve) is simply the vertical distance between the $\mathcal {C}_{\text{FRP}}$ value at that catastrophic threshold $\tau$ and 1 (in other words, $1-\mathcal {C}_{\text{FRP}}(\tau )$). For example, the percentage of households for whom OOP spending exceeds the $40\%$ threshold (area A on Fig. 1a) is shown by the vertical distance also labeled A (Fig. 1b). The percentage of households spending between 25 and $40\%$ of income is also shown (labeled B)^3^.

### Motivation

CHE estimates have been extensively used as summary measures of country performance on FRP. However, in some instances, national CHE point estimates may not capture some features of country performance on FRP. Here, we study four simulated datasets purposefully designed to highlight the relevance of building on CHE estimation with the study of distributional OOP share. Details on the simulated datasets are provided in Appendix A (online).

**Fig. 2:**
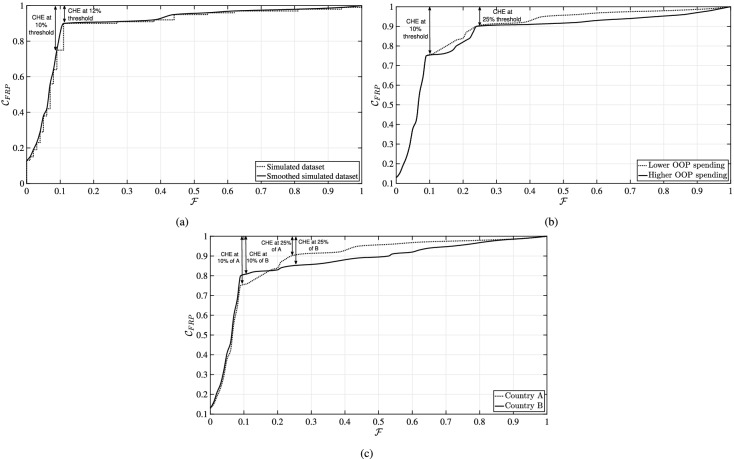
Illustrations of the motivation behind examining distributional OOP share, using three examples of CHE estimation. ** a** CHE is highly sensitive to the choice of the catastrophic threshold. ** b** CHE does not capture the magnitude of OOP payments. ** c** The ranking of country performance is subjective to the choice of the catastrophic threshold for CHE estimation. CHE: catastrophic health expenditures; OOP: out-of-pocket

The first dataset emphasizes the sensitivity of CHE computations to the selected threshold. The number of households whose $\mathcal {F}$ is greater than a specified threshold $\tau$ is usually determined, with $\tau$ being set to either 10$\%$, 25$\%$ or 40$\%$ [10, 11, 15]. The distinctive characteristic of this dataset is the high concentration of households with OOP expenditures constituting about $11\%$ of their income. When a $10\%$ threshold is applied, it is found that $25\%$ of the households would face CHE (vertical distance above the $\mathcal {C}_{\text{FRP}}$ curve at $\mathcal {F}=0.10$). However, if the threshold is slightly increased to $12\%$, the percentage of households facing CHE would drop from 25 to $10\%$ (vertical distance above the $\mathcal {C}_{\text{FRP}}$ curve at $\mathcal {F}=0.12$). The corresponding cumulative distribution $\mathcal {C}_{\text{FRP}}$ is shown (Fig. 2a). As can be seen, a mathematical discontinuity exists at $\mathcal {F}=0.11$ that leads to quite different CHE summary point estimates, even at those relatively close thresholds of 10 and $12\%.$^4^

Secondly, we highlight the non-sensitivity of CHE point estimates to the magnitude of OOP payments. We consider two datasets with the same proportion of households falling below the $10\%$ threshold ($75\%$ of households), the same proportion between the 10 and $25\%$ thresholds ($15\%$), and the same proportion of households above the $25\%$ threshold ($10\%$). Yet, in one dataset, households incur higher OOP payments than in the other dataset (Fig. 2b). Such differential magnitude in OOP payments would not be captured by estimating CHE headcounts^5^. For example, when a $25\%$ threshold is selected, both datasets would lead to the same estimations ($10\%$ of households facing CHE; Fig. 2b).

Estimates of CHE can often be used to compare countries with regard to their national performance on FRP provision. Because of the mathematical computation underlying CHE estimation, the resulting country rankings could highly depend on the chosen catastrophic threshold. For instance, take the OOP share cumulative distributions of two simulated countries (Fig. 2c): as we observe, the 10 and $25\%$ catastrophic thresholds would lead to diverging inconsistent views regarding which country is performing better. In fact, Hsu et al. [16] recently stated that interpretations of global comparisons based on a single threshold may not always be reliable in describing a country’s relative progress, and further recommended to measure country’s financial protection against CHE across a range of thresholds using a catastrophic incidence curve such as the one shown in Fig. 1b.

### Numerical index

In addition to possibly extracting any CHE estimate from the $\mathcal {C}_{\text{FRP}}$ curve (Fig. 1b), we derive an index that utilizes the curve’s features to measure the deviation or “distance” between two possible distributional OOP share states, say from an actual (current) state to a target state under given circumstances (e.g. financing policies).

**Fig. 3:**
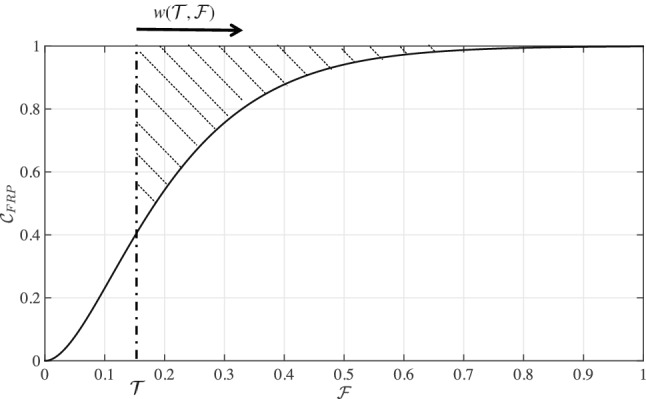
Illustrative example showing how the index can measure the deviation or “distance” between two OOP share states, an actual state and a target state. The dashed area corresponds to the departure of the existing OOP share state from a target of keeping OOP spending below $\mathcal {T}=15\%$ of income. $w(\mathcal {T},\mathcal {F})$ indicates weights that can be applied to $\mathcal {F}$ to augment the relative contribution of households at the right tail of the distribution. FRP: financial risk protection; OOP: out-of-pocket

We define a target $\mathcal {T}$ that aims to have all households bear OOP spending below a certain percentage $\mathcal {T}$ of income. To assess performance toward this target, a summary value representing the deviation of the country’s current state from the target state will be useful. In the illustrative example displayed (Fig. 3), the target would constrain $\mathcal {F}$ below $\mathcal {T}=15\%$. The deviation of the current state from target can be calculated via the $\mathcal {C}_{\text{FRP}}$ curve’s characteristics beyond the target state. Questions of interest will notably include: what is the existing range of $\mathcal {F}$ values? What is the slope of the curve beyond $\mathcal {F}=\mathcal {T}$? At what $\mathcal {F}$ value does the curve reach 1? The area enclosed between the $\mathcal {C}_{\text{FRP}}$ curve and the constant function $\overline{\mathcal {C}}_{\text{FRP}}=1$ can address all such questions. When this area equals 0, the target is achieved: no households would pay above $\mathcal {T}$ percentage of income. An increase in the area would translate into a greater deviation from the target state. The dashed area corresponds to the departure of the actual OOP share state from the targeted OOP share state (Fig. 3). As financial burden is reduced (say via increased coverage and subsidization of medical care), households would shift towards lower values of $\mathcal {F}$ causing the shaded area to decrease and eventually reaching 0.

We also consider the following two points. First, the index should be sensitive to the probability mass on the right tail of the cumulative distribution function $\mathcal {C}_{\text{FRP}}$ to emphasize the effect of high OOP spending relative to income. This can be easily done by applying weights (to $\mathcal {F}$) that increase with $\mathcal {F}$. Second, empirical data (on ranges of OOP spending relative to income) could be used to scale the index (e.g. to allow plausible value ranges the index can take) in order to increase the interpretability of the numerical values it yields.

The weighted area enclosed between the $\mathcal {C}_{\text{FRP}}$ curve and $\overline{\mathcal {C}}_{\text{FRP}}=1$ boundary can be expressed as:

1 $$\begin{aligned} A(\mathcal {T})=\int \limits _{\mathcal {T}}^{\mathcal {F}_{\text{max}}} w(\mathcal {T},\mathcal {F})\Big (1-\mathcal {C}_{\text{FRP}}(\mathcal {F})\Big ) d\mathcal {F}, \end{aligned}$$

where $\mathcal {F}_{\text{max}}$ is the maximum observed value of $\mathcal {F}$, and $w(\mathcal {T},\mathcal {F})$ is a weighting function that can augment the relative contribution of households at the right tail of the distribution (i.e. higher $\mathcal {F}$ values)^6^.

**Fig. 4:**
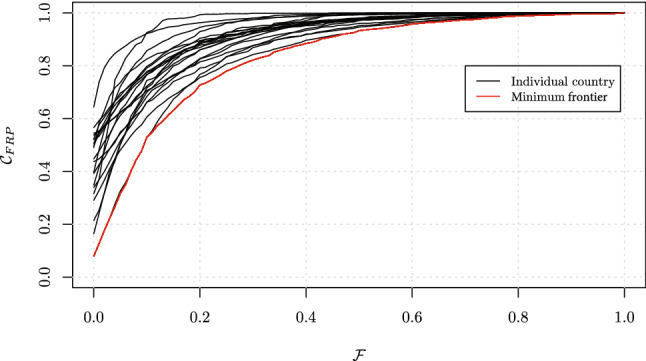
Graphical representation of the cumulative distribution $\mathcal {C}_{\text{FRP}}$ for 20 out of the 40 countries included in the World Health Surveys. $\mathcal {C}^{\text{min}}_{\text{FRP}}$ or the “minimum frontier” $\mathcal {C}_{\text{FRP}}$ value across all countries for every $\mathcal {F}$ value is shown in red

To improve the interpretability of the values yielded by the index, as an illustration, we apply it to the World Health Organization’s World Health Surveys (WHS) [13, 17, 18], and plot the cumulative distribution curve $\mathcal {C}_{\text{FRP}}$ for 40 illustrative countries (Fig. 4). The WHS was implemented over 2002–2004 across low-, middle- and high-income countries. It deployed a multistage sampling design to capture a nationally representative population. Households were asked to report total expenditures for a range of items over the last four weeks. This was summed to calculate total household spending, which we used as our measure of household consumption expenditures. We summed households’ 30-day OOP expenditures (annual spending divided by 12) on inpatient care, outpatient care, care from traditional providers, medicines, diagnostics and other health care costs (excluding health insurance reimbursements) to calculate monthly OOP health spending. Households also reported monthly total spending and monthly OOP health spending. Where the latter amounts were higher than the former calculated amounts, we used the latter reported totals. We divided OOP health spending by household consumption expenditures to derive empirical distributions of CHE (see below). We use such WHS data to construct a lower bound to the observable $\mathcal {C}_{\text{FRP}}$ curves. For every $\mathcal {F}$ value, the minimum $\mathcal {C}_{\text{FRP}}$ value among all WHS countries is extracted and serves to build the frontier $\mathcal {C}^{\text{min}}_{\text{FRP}}$, which represents a minimum across all countries (Fig. 4). Note that, evidently, $\mathcal {C}^{\text{min}}_{\text{FRP}}$ (cross-country “minimum frontier”) is highly sensitive to the data at hand (here the WHS data) and bears no specific health financing meaning.

Next, we apply Eq. (1) to $\mathcal {C}^{\text{min}}_{\text{FRP}}$ to estimate the maximum deviation possible at every $\mathcal {F}$ (i.e. the largest possible area). We then use those estimates to normalize our computed areas of interest (dashed areas, as on Fig. 3). The index $\mathcal {I}_{\text{FRP}}(\mathcal {T})$ can be expressed as:

2 $$\begin{aligned} \mathcal {I}_{\text{FRP}}(\mathcal {T})=\frac{\int \nolimits _{\mathcal {T}}^{\mathcal {F}_{\text{max}}} w(\mathcal {T},\mathcal {F})\Big (1-\mathcal {C}_{\text{FRP}}(\mathcal {F})\Big ) d\mathcal {F}}{\int \nolimits _{\mathcal {T}}^{\mathcal {F}_{\text{max}}} w(\mathcal {T},\mathcal {F})\Big (1-\mathcal {C}^{\text{min}}_{\text{FRP}}(\mathcal {F})\Big ) d\mathcal {F}}. \end{aligned}$$

As a result, $\mathcal {I}_{\text{FRP}}(\mathcal {T})$ can quantify the deviation from any chosen $\mathcal {T}$ on a 0 to 1 scale. Clearly, in Eq. (2), the denominator, which draws on $\mathcal {C}^{\text{min}}_{\text{FRP}}$ and normalizes the numerator, constrains the interpretability of cross-country comparisons within the data at hand (here the WHS data), and does not offer a normative view of what is a universally agreed $\mathcal {C}^{\text{min}}_{\text{FRP}}$ estimate. Evidently, other $\mathcal {C}^{\text{min}}_{\text{FRP}}$ estimates could be derived using alternative data sources (e.g. living standard surveys, household budget surveys, health expenditure and utilization surveys). Here, we pursue this normalization because we empirically observe $A(\mathcal {T})$ estimates (from Eq. (1)) to evolve in a much reduced space within the 0–1 rectangle of Fig. 4.

In what follows, for simplicity of exposition, the weights ($w(\mathcal {T},\mathcal {F})$) are set to be the horizontal distance between a point on the curve ($\mathcal {C}_{\text{FRP}}$) and the chosen $\mathcal {T}$: $w(\mathcal {T},\mathcal {F})=\mathcal {F}-\mathcal {T}$, which corresponds to household-level catastrophic overshoot. Many alternative formulations of the weighting function *w* could be used (see examples in [19–21]). For example, the weights could reflect attitudes to risk (e.g. via risk measures drawn from a constant relative risk aversion utility function); or one could use upper partial moments of the OOP share distribution with appropriately chosen risk aversion parameters as in Flores and O'Donnell [20]. The ensuing mathematical derivations would be further complicated without impacting the fundamental nature of our approach, and we leave the choice of normatively appealing weighting functions to the readers. In what follows, all simulations and results were generated with the software R Studio Version 1.1.453.

## Results

We discuss the features of the distributional OOP share approach, of $\mathcal {C}_{\text{FRP}}$ and $\mathcal {I}_{\text{FRP}}$, and their relevance to analyzing performance on FRP, with the application to WHS data to showcase the relevance of the approach.

**Fig. 5:**
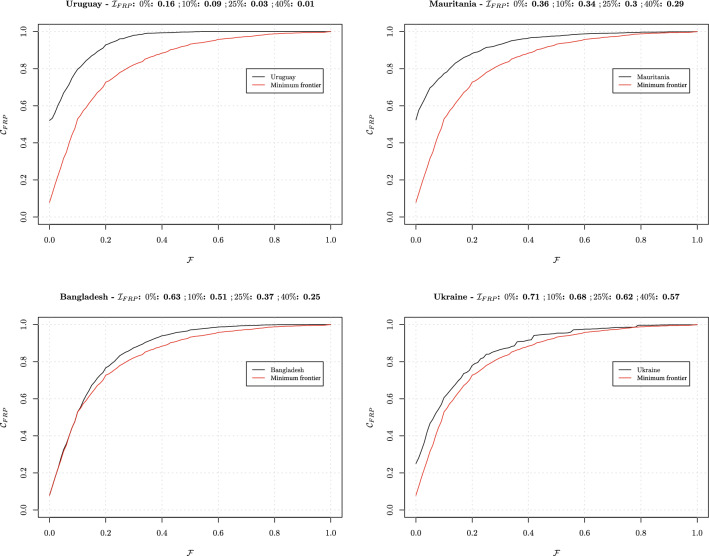
Cumulative distribution $\mathcal {C}_{\text{FRP}}$, and values of $\mathcal {I}_{\text{FRP}}$ index for four thresholds (0, 10, 25, and 40%): Uruguay, Mauritania, Bangladesh and Ukraine. Note: for illustrative graphical purposes, we added an “idealistic” 0% threshold to the commonly used thresholds of 10, 25, and 40%. Source: World Health Surveys

We compute and plot $\mathcal {C}_{\text{FRP}}$ for four contrasting countries: Uruguay, Mauritania, Bangladesh, and Ukraine (Fig. 5). For each country, we display the worst possible scenario, that is to say the minimum $\mathcal {C}^{\text{min}}_{\text{FRP}}$ (extracted from all countries in WHS data). We also compute the index ($\mathcal {I}_{\text{FRP}}$) for multiple targets that restrict OOP spending below either 0, 10, 25, or 40$\%$ of income (“income” = consumption expenditures computed from WHS in what follows). $\mathcal {I}_{\text{FRP}}$ represents the country deviation from achieving one target (we recall scores of 0 and 1 correspond to best and worst performances, respectively).

We observe that Uruguay performs best among the four countries as its $\mathcal {C}_{\text{FRP}}$ curve is the furthest away from $\mathcal {C}^{\text{min}}_{\text{FRP}}$ (red curve). For completeness, we also refer to the $\mathcal {I}_{\text{FRP}}$ values. For all four targets of OOP spending, the index values for Uruguay are the lowest, which indicates best performance. For example, when $\mathcal {T}=40\%$, Uruguay’s $\mathcal {I}_{\text{FRP}}$ is equal to 0.01, which indicates a situation where all households would pay less than $40\%$ of income on health. Mauritania would perform worse than Uruguay but better than Bangladesh. When comparing both countries with regards to their proximity in having all households pay less than $10\%$ of income, Mauritania would score better (0.34 vs. 0.51 for Bangladesh). However, $\mathcal {I}_{\text{FRP}}$ suggests that beyond $T=40\%$, Bangladesh would perform better than Mauritania (0.25 vs. 0.29), as households would be concentrated around higher $\mathcal {F}$ values in the case of Mauritania. These simple examples point to the usefulness of reporting $\mathcal {I}_{\text{FRP}}$ values (for varying thresholds *T*) alongside both CHE headcount and catastrophic overshoot estimates. For example, in Ukraine, examining $\mathcal {C}_{\text{FRP}}$ shows that $40\%$ of households would have OOP spending above a threshold of $10\%$, compared with $45\%$ of households in Bangladesh. This would suggest that Bangladesh would perform worse than Ukraine, while $\mathcal {I}_{\text{FRP}}$ values actually show that Bangladesh would be closer than Ukraine to a state where all households pay less than $10\%$ of income (0.51 vs. 0.68 for Ukraine) on health.

**Fig. 6:**
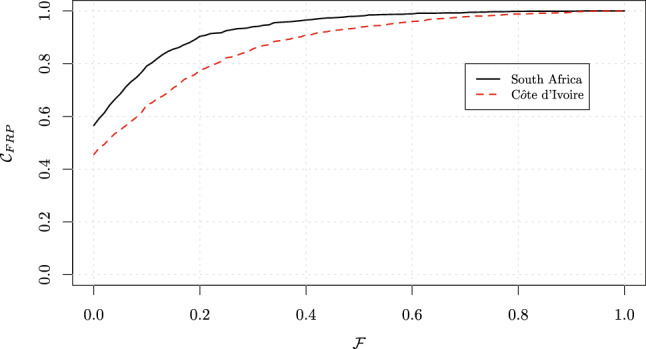
Cumulative distribution $\mathcal {C}_{\text{FRP}}$ of the ratio $\mathcal {F}$: South Africa and C$\hat{\text {o}}$te d’Ivoire. Scenario 1, where one country performance fully surpasses another. Source: World Health Surveys

Three different scenarios may arise across countries. Firstly (Scenario 1), one country can fully surpass another: see, for example, the comparison of South Africa with C$\hat{\text {o}}$te d’Ivoire, where the countries’ $\mathcal {C}_{\text{FRP}}$ curves do not cross (Fig. 6). Here, South Africa performs better across all $\mathcal {F}$ values as can be seen from its higher $\mathcal {C}_{\text{FRP}}$ curve. CHE headcount estimates and $\mathcal {I}_{\text{FRP}}$ values for different thresholds (10, 25, and 40%) are computed (Table 1) for Scenario 1. As expected, South Africa performs better across all thresholds (Table 1).

**Table 1:** CHE and $\mathcal {I}_{\text{FRP}}$ index values for South Africa and C$\hat{\text {o}}$te d’Ivoire for different thresholds $\mathcal {T}$ (10, 25, and 40%)

Threshold	10%		25%		40%	
Metric	CHE	$\mathcal {I}_{\text{FRP}}$	CHE	$\mathcal {I}_{\text{FRP}}$	CHE	$\mathcal {I}_{\text{FRP}}$
South Africa	20%	0.28	9%	0.24	4%	0.21
C$\hat{\text {o}}$te d’Ivoire	40%	0.83	20%	0.85	10%	0.86

CHE: catastrophic health expenditures. Source: World Health Surveys

**Fig. 7:**
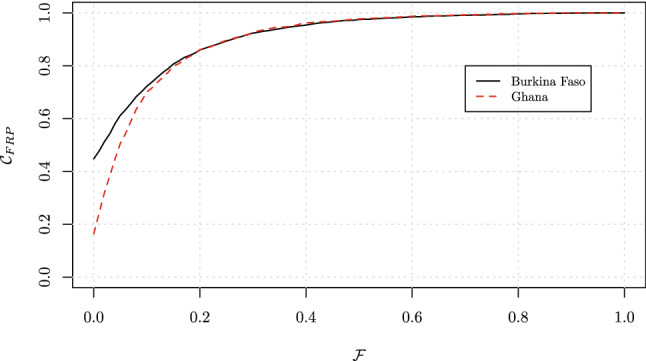
Cumulative distribution $\mathcal {C}_{\text{FRP}}$ of the ratio $\mathcal {F}$: Burkina Faso and Ghana. Scenario 2, where countries’ performance converge. Source: World Health Surveys

Secondly (Scenario 2), the country cumulative distribution curves $\mathcal {C}_{\text{FRP}}$ converge to the same values: see, for example, the cases of Burkina Faso and Ghana (Fig. 7). The CHE estimates extracted from $\mathcal {C}_{\text{FRP}}$ at a catastrophic threshold of $10\%$ show that Burkina Faso would perform better than Ghana. However, when such CHE estimates are supplemented by $\mathcal {I}_{\text{FRP}}$ index values which capture the distribution of households across values of $\mathcal {F}$ beyond $\mathcal {T}=10\%$, we observe similar performance between the two countries (Table 2). This implies that although Burkina Faso would have fewer households experiencing CHE at low thresholds of $10\%$ and below (see the divergence of the two curves for low OOP budget share values), both countries would have similar performance when the distribution of all OOP budget share is considered. This demonstrates the usefulness of supplementing CHE estimates with an index that can quantify the deviation from a chosen target $\mathcal {T}$.

**Table 2:** CHE and $\mathcal {I}_{\text{FRP}}$ index values for Burkina Faso and Ghana for different thresholds (10, 25, and 40%)

Threshold	10%		25%		40%	
Metric	CHE	$\mathcal {I}_{\text{FRP}}$	CHE	$\mathcal {I}_{\text{FRP}}$	CHE	$\mathcal {I}_{\text{FRP}}$
Burkina Faso	30%	0.39	10%	0.32	4%	0.30
Ghana	36%	0.39	10%	0.32	3%	0.30

CHE: catastrophic health expenditures. Source: World Health Surveys

**Fig. 8:**
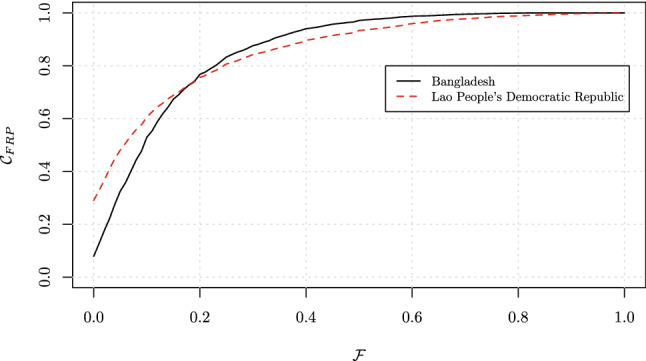
Cumulative distribution $\mathcal {C}_{\text{FRP}}$ of the ratio $\mathcal {F}$: Bangladesh and Lao People’s Democratic Republic (PDR). Scenario 3, where countries’ performance curves cross. Source: World Health Surveys

Lastly (Scenario 3), country $\mathcal {C}_{\text{FRP}}$ curves would cross each other. Such a situation stresses the importance of a density metric, as can be seen with the comparison between Bangladesh and Lao People’s Democratic Republic (PDR) (Fig. 8). The point of intersection between the two curves represents the threshold value above which a similar proportion of households in the two countries would face CHE. In this example, around 45$\%$ of households in both Burkina Faso and Lao PDR would face CHE with a threshold of $10\%$. Below this threshold, CHE values show that Lao PDR would perform better (higher $\mathcal {C}_{\text{FRP}}$ curve), while above that threshold (say $25\%$) Bangladesh would perform better. Such dependence on thresholds could be resolved by $\mathcal {I}_{\text{FRP}}$ (Table 3). For instance, at $\mathcal {T}=10\%$, even though CHE would be the same in both countries, $\mathcal {I}_{\text{FRP}}$ values would be very different, showing that Bangladesh in fact would perform much better. This is because the sensitivity of $\mathcal {I}_{\text{FRP}}$ to the probability mass distribution can capture the fact that households in Lao PDR would experience much higher OOP spending relative to income.

**Table 3:** CHE and $\mathcal {I}_{\text{FRP}}$ index values for Bangladesh and Lao People’s Democratic Republic (PDR) for different thresholds (10, 25, and 40%)

Threshold	10%		25%		40%	
Metric	CHE	$\mathcal {I}_{\text{FRP}}$	CHE	$\mathcal {I}_{\text{FRP}}$	CHE	$\mathcal {I}_{\text{FRP}}$
Bangladesh	50%	0.51	15%	0.36	5%	0.24
Lao PDR	39%	0.90	20%	0.90	10%	0.88

CHE: catastrophic health expenditures. Source: World Health Surveys

## Discussion

We introduced in this paper a density analysis of health OOP budget share ($\mathcal {D}_{\text{FRP}}$) as a stepping stone toward assessing the performance of national health systems on FRP and to enable better description of the impact of health interventions and financing policies on FRP. $\mathcal {D}_{\text{FRP}}$ builds on the routinely used metric of CHE (and associated computation of catastrophic payment overshoot or gap) and is composed of a cumulative distribution including a graphical representation ($\mathcal {C}_{\text{FRP}}$), and a numerical index ($\mathcal {I}_{\text{FRP}}$), which can jointly summarize country performance on FRP and measure its distance (or gap) from achieving certain targets. Also, we have used the World Health Organization’s World Health Surveys to include examples of multiple countries in order to showcase the application of our proposed methods; such illustrative examples were not chosen to fully investigate the FRP states of the selected countries nor to interpret country-specific findings.

Such an approach can prove useful in tracking progress toward UHC [1, 4, 22]. Importantly, given the increasing attention in the post-2015 agenda to measures of inequality reductions, FRP and poverty reduction, our examination of the full distribution of OOP budget share is timely in proposing to report comprehensively on the density of lack of FRP in countries. Therefore, $\mathcal {D}_{\text{FRP}}$ would well supplement the current cross-national estimations of CHE headcounts [11] and mean catastrophic overshoots. Our approach provides flexibility for computing a variety of thresholds of potential interest to policymakers. A specific threshold can be computed that closely aligns with country-level policies (e.g. local social protection programs); and the overall density analysis of health OOP budget share may inform policymakers concerned with discontinuities and disaggregated impacts associated with certain interventions, programs, or taxes, etc.

Nevertheless, our approach presents a number of important limitations. First, as in the computation of other FRP metrics (e.g. CHE, IHE), $\mathcal {D}_{\text{FRP}}$ is heavily reliant on the available data at hand, including the household surveys on OOP spending and consumption expenditures that can vary significantly across countries with varying survey instruments and reporting quality standards (e.g. recall bias), which can hamper cross-country comparability [23].

Second, while potentially comprehensive across the whole population, analysts and decision-makers would still need to interpret the levels and shapes of $\mathcal {C}_{\text{FRP}}$ estimated, in other words which shapes of cumulative density curves should be preferred and how different shapes may correspond to different states and types of financing such as high OOP costs due to a large private sector or high copayments, the absence of specific health services (e.g. poor availability of non-communicable disease services in many low- and middle-income countries), the absence of OOP spending due to unaffordability to seek care, or difficulties in access, etc. In this respect, future work with cross-country analyses will enable defining a typology of $\mathcal {C}_{\text{FRP}}$ distributions and $\mathcal {I}_{\text{FRP}}$ values, which can help quantitatively define certain desirable features of national health systems (e.g. pro-poor vs. universal public finance). Likewise, applying $\mathcal {D}_{\text{FRP}}$ can help measure the impact of certain policies on FRP to then highlight when efficient investments toward FRP improvement in countries could be made.

Third, our approach heavily uses densities and distributions in order to supplement the traditional FRP metrics with expanded summary information. An approach such as ours that relies on the accuracy of the underlying FRP distributions and empirical data may be more sensitive to measurement errors and sampling design in data collection. Therefore, an analysis that compares such potential sensitivity of our method with that of the traditional metrics is a topic of future work.

Fourth, the WHS surveys were used to constrain the numerical index $\mathcal {I}_{\text{FRP}}$ between 0 and 1 in order to increase its interpretability. While other surveys could have been used (e.g. Living Standards Measurement Surveys) and might have affected the results slightly, we used WHS for illustrative purposes only, because of their extensive usage in the literature. Because the FRP state of countries has been improving since the WHS’s 2002–2004 time window, the “minimum frontier” constructed through the WHS surveys to normalize $\mathcal {I}_{\text{FRP}}$ in this paper might still be relevant today.

In summary, building on computations of CHE headcounts and associated mean catastrophic payment overshoots, examining the full density of OOP budget share can aid analysts in reporting on the features of FRP performance of national health systems along with their progress toward UHC. It can prove to be especially practical in analyzing the impact of health policy on population-level FRP (e.g. public vs. private financing of health services, copayments for social health insurance schemes, etc.). This could be a stepping stone toward comprehensively synthesizing the level and distribution of financial risk protection, hence the performance of health systems, across countries globally.

## Electronic Supplementary Material

Below is the link to the electronic supplementary material.

Supplementary material 1 (PDF 79 kb)

## Data Availability

WHS data can be downloaded from WHO website upon permission.

## Footnotes

1 This graphical representation was chosen for illustration purposes only. It corresponds to a gamma distribution with shape parameter $k=2$ and scale parameter $\theta =11$ over the range $0\le \mathcal {F}\le 100\%$.

2 This is consistent with the inverse cumulative distribution function of the OOP budget share used by Wagstaff and van Doorslaer [6] to illustrate measures of catastrophic payments.

3 Note that a possible stepping stone to Fig. 1b can be constructed by calculating the percentage of households facing CHE for a variety of catastrophic thresholds; such CHE estimates can be plotted using ADePT software [24].

4 This discontinuity issue is also discussed by Flores and O'Donnell [20] who developed a metric of catastrophic medical expenditure risk to overcome this limitation.

5 Note that separate additional analyses such as the computation of catastrophic overshoots could be conducted [6, 24].

6 Weights would depend on both $\mathcal {T}$ and $\mathcal {F}$, and different mathematical specifications could be used for $w(\mathcal {T},\mathcal {F})$. The unweighted formulation of Eq. (1) is related to the mean catastrophic overshoot or gap introduced by Wagstaff and van Doorslaer [6] and to the first order upper partial moment of the OOP budget share distribution discussed by Flores and O'Donnell [20].
