# Supplementary material for: Examining the density in out-of-pocket spending share in the estimation of catastrophic health expenditures
Source: Eur J Health Econ. 2021 Aug 6;23(5):903–12. doi: 10.1007/s10198-021-01316-x (PMC9170678; doi:10.1007/s10198-021-01316-x)
Supplement: Supplementary file 1 — Supplementary material 1 (PDF 79 kb) [file 10198_2021_1316_MOESM1_ESM.pdf]

Appendices

A. Simulated datasets used in subsection "Motivation"

Four household datasets were designed to simulate the distributional FRP metric under varying illustrative cases. Each dataset represents a hypothetical FRP state and spans 100 households. The datasets are labeled A to D and are detailed below. The numbers indicated on figure A.1 correspond to the percentage of income spent out of pocket on health per household.

We first construct dataset A by specifying the number of households below the 10% catastrophic threshold (color-coded gray), between the 10 and 25% thresholds (color-coded yellow), and above the 25% threshold (color-coded red). We then use a random number generator that outputs OOP spending within the specified constraints. All other datasets (B to D) are generated by simple modifications to dataset A.

In order to construct dataset B, we use dataset A and only modify the OOP values between the 10 and 25% thresholds by setting them equal to 11%. This dataset is used to emphasize the dependence of CHE estimates on the catastrophic threshold selected.

In order to construct dataset C, we use dataset A and only vary the magnitude of OOP spending for households for which OOP spending exceeds the 10% threshold. As can be seen, the proportion of households remains intact, but OOP spending values in dataset C that are yellow and red are higher than those of dataset A. This dataset is used to show the importance of capturing the magnitude of OOP spending.

Dataset D results from dataset A by modifying the OOP values between the 10 and 25% thresholds and allowing the distribution of households within the 10 and 25% thresholds to change (note the variations in colors). This dataset is used to show inconsistency in ranking different FRP states using CHE at different thresholds.

A	B	C	D
17	11	22	6
15	11	18	2
18	11	21	4
12	11	14	9
21	11	23	1
20	11	21	20
21	11	24	21
13	11	16	13
11	11	19	11
24	11	24	24
21	11	23	32
24	11	24	44
22	11	23	65
16	11	18	86
15	11	17	72
81	81	90	81
44	44	60	44
27	27	40	60
44	44	54	44
94	94	97	94
100	100	100	100
44	44	78	77
62	62	86	62
54	54	94	54
38	38	70	53
7	7	7	7
9	9	9	9
9	9	9	9
5	5	5	5
2	2	2	2
7	7	7	7
4	4	4	4
9	9	9	9
7	7	7	7
2	2	2	2

Simulated datasets.

5	5	5	5
5	5	5	5
4	4	4	4
6	6	6	6
5	5	5	5
3	3	3	3
0	0	0	0
9	9	9	9
1	1	1	1
7	7	7	7
5	5	5	5
0	0	0	0
0	0	0	0
9	9	9	9
0	0	0	0
5	5	5	5
4	4	4	4
7	7	7	7
5	5	5	5
5	5	5	5
1	1	1	1
9	9	9	9
8	8	8	8
7	7	7	7
2	2	2	2
8	8	8	8
8	8	8	8
9	9	9	9
9	9	9	9
0	0	0	0
8	8	8	8
7	7	7	7
7	7	7	7
2	2	2	2
0	0	0	0
3	3	3	3

Simulated datasets (continued).

0	0	0	0
8	8	8	8
4	4	4	4
6	6	6	6
7	7	7	7
7	7	7	7
4	4	4	4
6	6	6	6
7	7	7	7
0	0	0	0
3	3	3	3
8	8	8	8
0	0	0	0
7	7	7	7
9	9	9	9
9	9	9	9
3	3	3	3
0	0	0	0
0	0	0	0
0	0	0	0
4	4	4	4
5	5	5	5
8	8	8	8
0	0	0	0
8	8	8	8
7	7	7	7
6	6	6	6
9	9	9	9
7	7	7	7

Simulated datasets (continued).

Figure A.1: Display of the four simulated datasets used in subsection "Motivation": $n = 100$ households per dataset. Every row corresponds to a household, and every numerical entry is the percentage of household income spent on health out of pocket. Fractions below 10, between 10 and 25 and above 25% are color-coded by grey, yellow, and red, respectively.
